# ﻿Identification key to and checklist of the Swedish Phlaeothripidae (Thysanoptera)

**DOI:** 10.3897/zookeys.1096.83011

**Published:** 2022-04-18

**Authors:** Emma Wahlberg, Carl-Axel Gertsson

**Affiliations:** 1 Department of Zoology, Swedish Museum of Natural History, P.O. Box 50007, SE-104 05 Stockholm, Sweden Department of Zoology, Swedish Museum of Natural History, Stockholm Sweden; 2 Murarevägen 13, SE-227 30 Lund, Sweden Unaffiliated Lund Sweden

**Keywords:** Distribution, first record, identification, morphology, taxonomy, Thrips, Tubulifera

## Abstract

The Swedish fauna of thrips (Thysanoptera) in the family Phlaeothripidae consists of 49 species. A key to the species of Phlaeothripidae found in Sweden is provided. One species is recorded as new for the country, and 10 new regional records are presented. A checklist of all Swedish tubuliferan species with regional distributions is also given.

## ﻿Introduction

**Figure 1.**
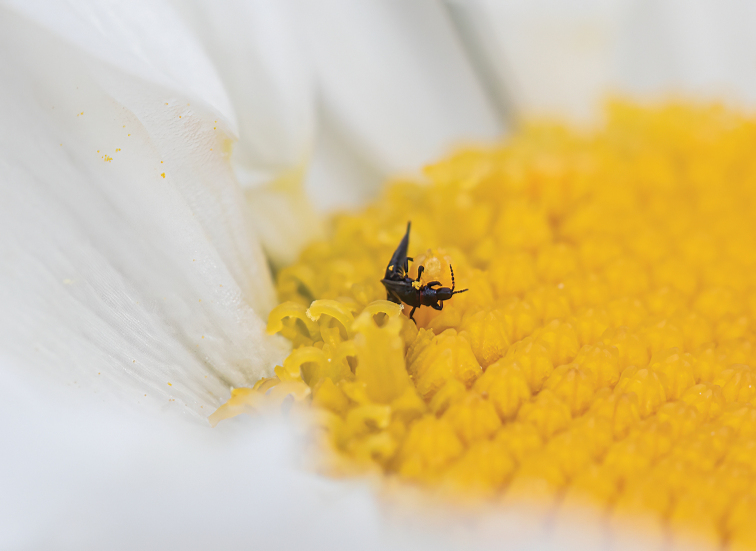
*Haplothripsleucanthemi* in flower of *Leucanthemumvulgare*.

Thysanoptera Haliday, 1836, more commonly known as thrips, are minute insects which are often not longer than 3 mm; larger species may reach 5 mm in size. Thrips have caught attention not only from researchers but also from the commercial and private sector, due to their impact as pests in agriculture (Paine 1992) and even as invasive species (Held et al. 2005; Boyd and Held 2006). The group least studied in Sweden is the family Phlaeothripidae Uzel, 1895. Some species are found in flowers, e.g., in the genus *Haplothrips* Amyot & Serville, 1843 (Fig. 1), but most of the known species in Sweden are found in soil, leaf litter, and decaying wood.

The research regarding Palaearctic taxa is scarce. Only a few regional checklists have been published in recent years, and the most relevant identification keys focus on the species in Great Britain (Mound et al. 1976, 2018; Kirk 1996). In recent years the fauna of Poland, a region with a previously similarly understudied thrips fauna, has been studied more extensively, which has led to a large gain in both taxonomic and ecological knowledge (Kucharczyk and Zawirska 1994; Kąkol and Kucharczyk 2004; Kucharczyk 2004; Kucharczyk and Kucharczyk 2008; Dubovský et al. 2010; Kucharczyk and Wyrozumski 2015). Most of the knowledge of Swedish taxa is based on older identification literature, e.g., Ahlberg (1926), Mound et al. (1976), and Kirk (1996), often not specific for Scandinavian conditions. A few papers have been published reporting new species at irregular intervals, reporting sporadic observations (Qvick 1977; Vasiliu-Oromulu et al. 2000; Kobro 2011; Sörensson 2012; Gertsson 2015a; Gertsson and Fägerström 2017). Kobro and Rafoss (2006) produced a key to the genus *Hoplothrips* in Norway, and Kobro (2013) produced an identification key to Norwegian thrips in general but only covered the most common and for amateurs easily distinguished species. The overlaps of the distributions the of Swedish and Norwegian species is currently not known, and no identification key to the Swedish fauna exists. Gertsson (2015b) provided a checklist of Nordic thrips. However, this was based only on previously collected specimens in museum collections. Recently new records to the fauna were made from freshly collected material, with a total of 5 new species for Sweden and several new regional records (Gertsson and Fägerström 2017; Gertsson 2021; Gertsson et al. 2022). In this paper we update the Swedish checklist of the family Phlaeothripidae and provide an identification key to the species with photographic illustrations.

## ﻿Material and methods

For this study we have examined representative specimens from the collections of The Swedish Museum of Natural History, Sweden (**NHRS**), Lund Museum of Zoology (**MZLU**), Sweden, Forschungsinstitut und Naturmuseum Senckenberg (**SMF**), Germany, and the private collections of Sverre Kobro and Manfred R. Ulitzka. In addition, newly collected material has been used, prepared on slides with Euparal according to the method outlined in Kobro (2013). The exception to this method is the preservation prior to maceration and the maceration step, where in this study fresh material has been stored in 80% ethanol prior to DNA extraction. Maceration has thereafter been carried out during DNA extraction before preparation on microscopic slides. This method has successfully been used for one-step DNA extraction and maceration for small insect specimens (Wahlberg and Johanson 2018; Wahlberg 2019). DNA extract is stored at the NHRS for further studies. The material was examined and photographed using manual focus stacking on Nikon Eclipse 80i and Swift 380T microscopes, with Nikon DS-Fi1 and Swift SC1003 cameras. Photos were automatically aligned and stacked using Helicon Focus 8.0.4 and Swift Imaging 3.0, and edited and finalized in Adobe Photoshop CC 23.2.0. The distributional data are provided on county level. The material collected and preserved during this project is deposited at the NHRS.

### ﻿Swedish faunistic provinces and abbreviations

**Figure 2.**
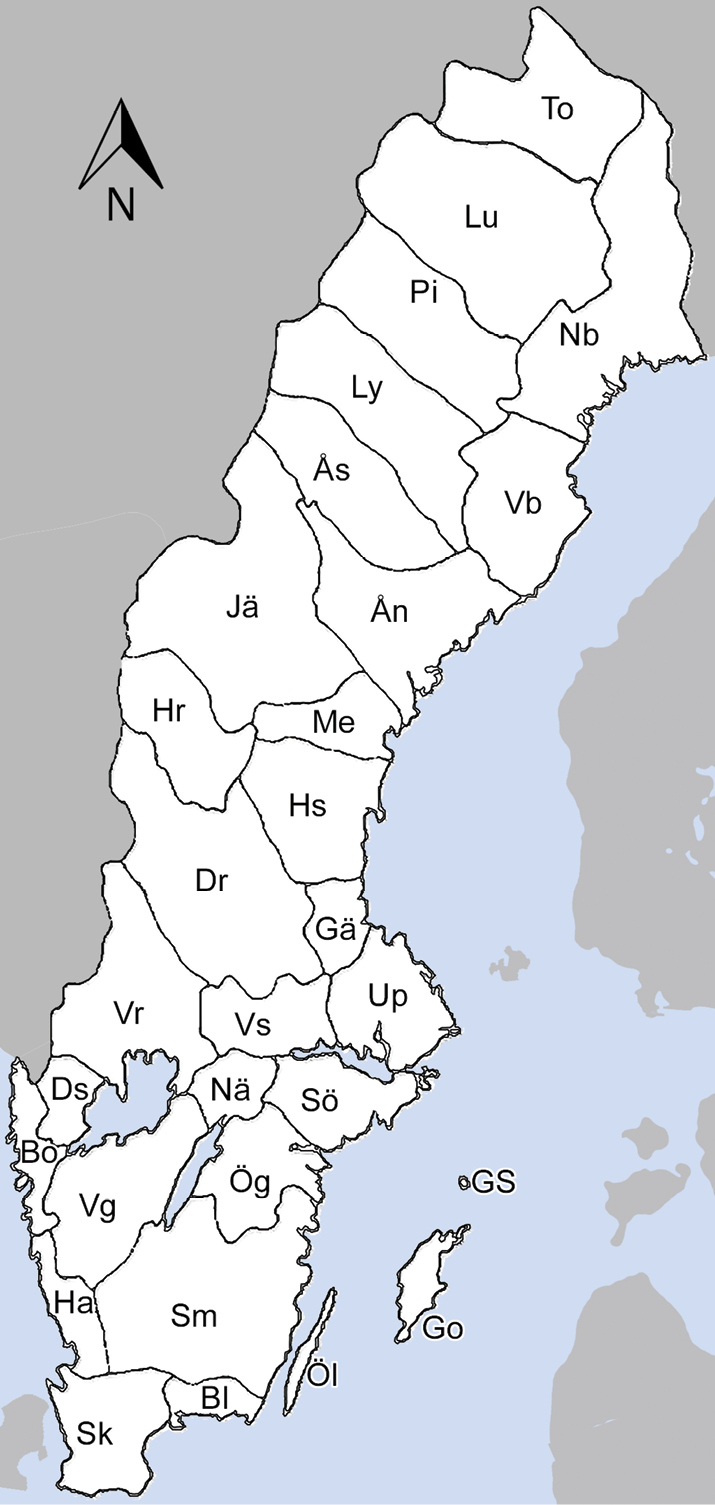
Map of Swedish faunistic provinces.

Sweden is traditionally divided in to faunistic provinces, most based on historical cultural regions overlapping with administrative counties (Fig. 2). They are in the checklist and map abbreviated as below, from south to north.

**Sk** Skåne

**Bl** Blekinge

**Ha** Halland

**Sm** Småland

**Öl** Öland

**Go** Gotland

**GS** Gotska Sandön

**Ög** Östergötland

**Vg** Västergötland

**Bo** Bohuslän

**Ds** Dalsland

**Nä** Närke

**Sö** Södermanland

**Up** Uppland

**Vs** Västmanland

**Vr** Värmland

**Dr** Dalarna

**Gä** Gästrikland

**Hs** Hälsingland

**Me** Medelpad

**Hr** Härjedalen

**Jä** Jämtland

**Ån** Ångermanland

**Vb** Västerbotten

**Nb** Norrbotten

**Ås** Åsele lappmark

**Ly** Lycksele lappmark

**Pi** Pite lappmark

**Lu** Lule lappmark

**To** Torne lappmark

### ﻿Characters

**Figure 3.**
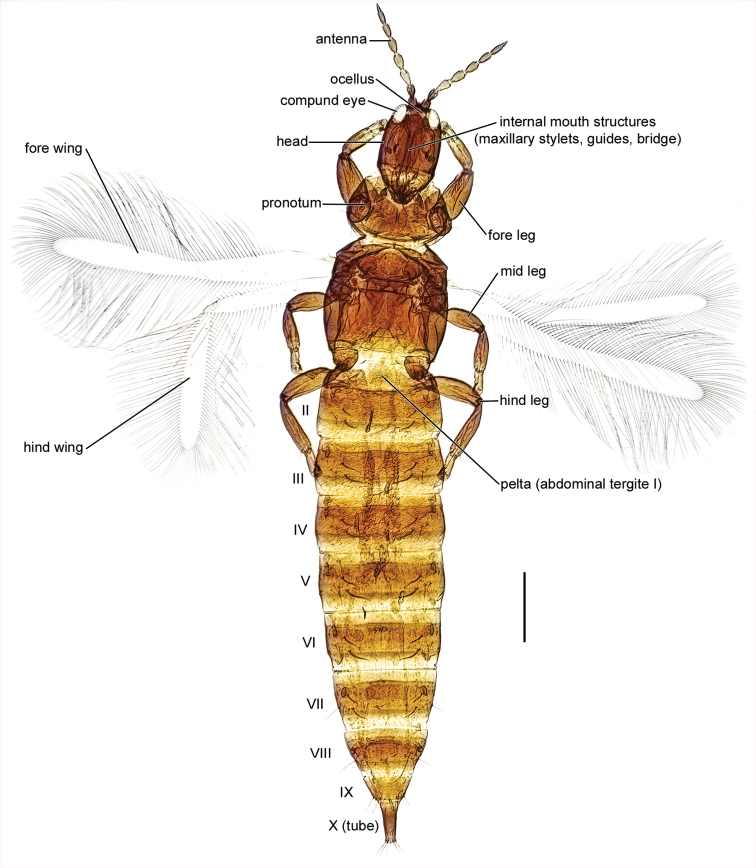
Habitus of *Haplothripsutae*, dorsal view. Roman numbers indicate abdominal segment number. Scale bar: 100 μm.

**Figure 4.**
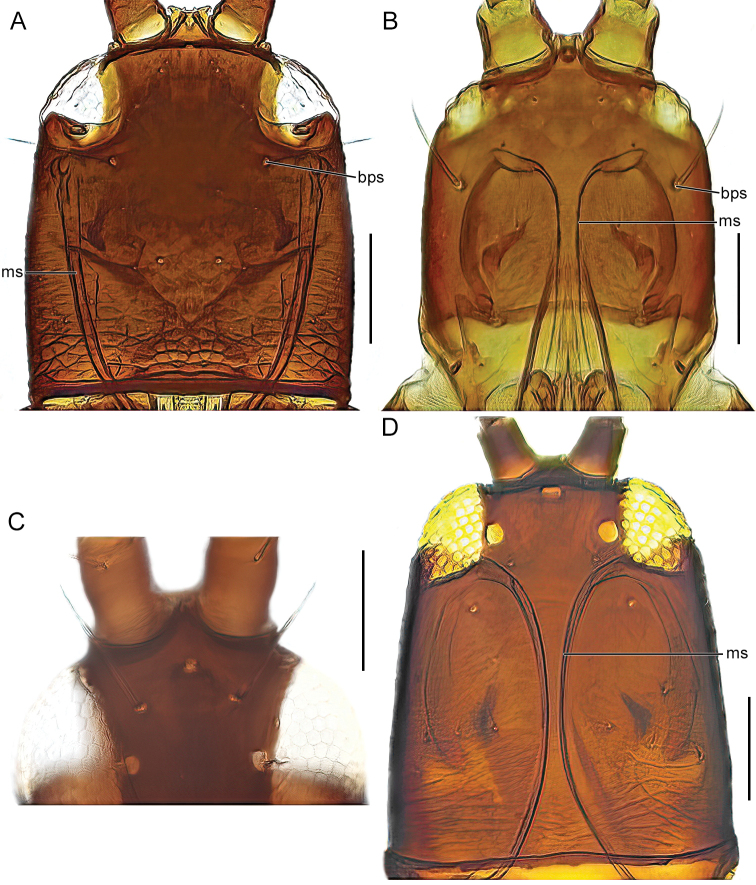
head, dorsal view **A***Bolothripsicarus***B***Hoplothripscarpathicus***C***Bacillothripsnobilis* (ocellar triangle) **D***Cryptothripsnigripes*. Abbreviations: bps: base of postocular seta, ms: maxillary stylets. Scale bars: 100 μm.

The identification key is intended to be used for adult specimens, both females and males in various life stages and both winged and micropterous forms. For this reason, some species that express great intraspecific variation it is possible to find one species at several locations in the key (indicated by “[part]”). In Thysanoptera the most important morphological characters for species identification include antennal shape, presence, shape, and length of setae, structure of mouth parts, and measurements of segments (Fig. 3). This always requires high magnification and specimen preparation. Large setae may be blunt, expanded (Fig. 10G), or acute at apex, and care need to be taken in preparation for avoiding collapse of expanded apices. The antennal segments often carry sensory organs in the shape of large trichomes, sense cones. These are more robust and broader than bristles that they might be confused with (Fig. 6A–C). Maxillary stylets are parts of the feeding apparatus and can be seen in macerated specimens (Fig. 4A, B), and the width and distance of the stylets and presence or absence of the median extension called maxillary bridge are used for separation of subfamilies and species groups. The last abdominal segment, segment X, may be either tapering and longitudinally divided (in most of the Thysanoptera families) or complete and tube-shaped. The latter being one of the defining characters of the family Phlaeothripidae (Fig. 3) and is in the key only referred to as the tube. Comprehensive and detailed descriptions of the anatomy and morphology of Thysanoptera are provided in Schliephake and Klimt (1979) and Moritz (2006).

## ﻿Taxonomy

### ﻿ Phlaeothripidae

Taxon classificationAnimaliaThysanopteraPhlaeothripidae

Uzel, 1895

855FFAD8-E8C6-5F59-86D9-E47BFFB1FBBF

#### Diagnosis.

**Figure 5.**
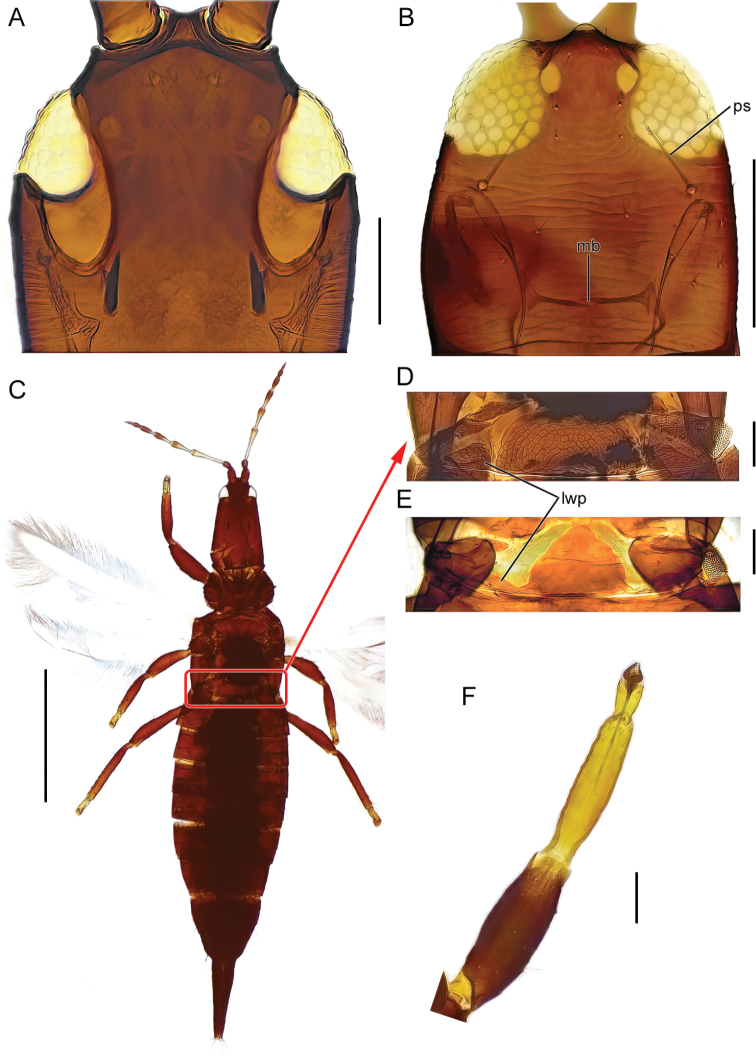
**A, B** head, dorsal view **A***Bolothripsdentipes* (ventral margins of eyes also visible) **B***Haplothripssenecionis***C** habitus, dorsal view, *Bacillothripsnobilis***D, E** pelta **D***B.nobilis***E***Megalothripsbonanni***F** fore leg, *Megathripslativentris*. Abbreviations: mb: maxillary bridge, ps: postocular setae, lwp: lateral wings of pelta. Scale bars: 100 μm (**A, B, D, E**), 1 mm (**C**).

The last abdominal segment, segment X, tubular in both males and females (Figs 3, 5C, 7A, B), without longitudinal division and without saw-like ovipositor. In macropterous forms fore wings without longitudinal veins and surface without microtrichia (Fig. 3). Wing fringes not on sockets but embedded into the wing membrane. Wing retaining setae present in all (European) macropterous species.

#### Notes.

There are about 3,700 known species of Phlaeothripidae in the world (Mound and Tree 2020; ThripsWiki 2022). Most of these species are described from tropical and subtropical areas. In Sweden 49 species are known. The Phlaeothripidae are diverse in their biology; feeding on decaying matter, pollen, fungal spores and hyphae, and prey, and sometimes expressing polymorphism and sociality (Kirk 1996; Mound 2004).

### ﻿Key to the species of Phlaeothripidae from Sweden

**Table:** 

1	Maxillary stylets broad, at least 5 μm wide (twice as wide as base of postocular setae) (Fig. 4A)	**1 (Idolothripinae)**
–	Maxillary stylets slender, less than 5 μm wide (Fig. 4B)	**7 (Phlaeothripinae)**
2 (1)	Anterior margin of ocellar triangle with long setae (Fig. 4C); large and dark species with elongated head	**3**
–	Setae at anterior margin of ocellar triangle short or absent (Fig. 4D)	**5**
3 (2)	Lateral wings of pelta (abdominal tergite I) slender (Fig. 5E)	***Megalothripsbonanni***
–	Lateral wings of pelta triangular (Fig. 5D)	**4**
4 (2)	Tarsi pale and tibiae brown (Fig. 5C)	***Bacillothripsnobilis***
–	Tarsi and tibiae yellow (Fig. 5F)	***Megathripslativentris***
5 (2)	Eyes ventrally elongated (Fig. 5A)	**6**
–	Eyes ventrally not elongated	**7**
6 (5)	Body (excluding antennae, legs, and wings) brown with yellow pronotum and yellow transverse band on metanotum	***Bolothripsbicolor***
–	Body uniformly brown	***Bolothripsdentipes***
7 (5)	Maxillary stylets close together, meeting or almost meeting medially (Fig. 4D)	***Cryptothripsnigripes***
–	Maxillary stylets widely separated, at least half of head width apart (Fig. 4A)	***Bolothripsicarus***
8 (1)	Maxillary bridge present (Fig. 5B)	**9**
–	Maxillary bridge absent (Fig. 4B)	**28**
9 (8)	Antennal segment IV with 2 sense cones (Fig. 6A)	***Xylaplothripsfuliginosus***
–	Antennal segment IV with 3 or 4 sense cones (Fig. 6B, C)	**10 (*Haplothrips*)**
10 (9)	Antennal segment III without sense cones (cf. Fig. 9E)	***Haplothripsminutus***
–	Antennal segment III with at least 1 sense cone	**11**
11 (10)	Antennal segment III with 1 sense cone	**12**
–	Antennal segment III with 2 sense cones	**14**
12 (11)	Postocular setae expanded or bluntly pointed (Fig. 6D)	***Haplothripssubtilissimus***
–	Postocular setae acute (Fig. 6E)	**13**
13 (12)	Tube more than 2.3 times longer than wide (Fig. 7A)	***Haplothripsalpester*** [part]
–	Tube less than 2.3 times longer than wide (Fig. 7B)	***Haplothripsaculeatus***
14 (11)	Postocular setae short, not longer than the width of the eye (Fig. 6F)	**15**
–	Postocular setae well developed and long (Fig. 7C)	**18**
15 (14)	Setae S1 on tergite IX blunt apically (Fig. 7D)	**16**
–	Setae S1 on tergite IX acute	**17**
16 (15)	Antennal segment IV yellow at base (Fig. 7E)	***Haplothripsleucanthemi***
–	Antennal segment IV completely brown (Fig. 7F)	***Haplothripspropinquus***
17 (15)	Both anteromarginal and anteroangular setae stout and at least twice as long as discal setae (Fig. 7G)	***Haplothripsalpicola***
–	Anteromarginal setae minute, anteroangular setae sometimes longer but not as stout	***Haplothripsangusticornis***
18 (14)	Setae S1 on tergite IX blunt apically (cf. Fig. 7D)	**19**
–	Setae S1 on tergite IX acute	**21**
19 (18)	Postocular setae acute (Fig. 7H)	***Haplothripstritici*** [part]
–	Postocular setae bluntly pointed (Fig. 5B)	**20**
20 (19)	Maxillary stylets one 1/3–1/4 of head width apart (Fig. 5B); tibia I brown	***Haplothripssenecionis***
–	Maxillary stylets about 1/5 of the head width apart (Fig. 7C); tibia I yellow apically	***Haplothripsstatices***
21 (18)	Distal cilia of fore wings with barbs (in high magnification), in lower magnification visible as a rough or frizzled surface (Fig. 8A)	***Haplothripssetiger***
–	Distal cilia of fore wings smooth	**22**
22 (21)	Postocular setae bluntly pointed (Fig. 8B)	**23**
–	Postocular setae acute (Fig. 7H)	**24**
23 (22)	Maxillary stylets about a fourth of head width apart (Fig. 8B); tibia I yellow but brown basally	***Haplothripsverbasci***
–	Maxillary stylets about half of head width apart (Fig. 8C); tibia I wholly yellow	***Haplothripsacanthoscelis***
24 (22)	Maxillary stylets close together, almost meeting medially (Fig. 8D)	***Haplothripsutae***
–	Maxillary stylets at least a third of head width apart (Fig. 4E)	**25**
25 (24)	Maxillary stylets half of head width apart (cf. Fig. 8C)	***Haplothripsdistinguendus***
–	Maxillary stylets 0.3–0.4 of head width apart (Fig. 7H)	**26**
26 (25)	Antennal segments III–IV, sometimes also V–VI, brown with yellow base, segments VII–VIII brown (Fig. 8E)	***Haplothripshukkineni***
–	Antennal segment III shaded yellow to light brown, IV–VII brown (Fig. 8F)	**27**
27 (26)	Anteromarginal setae short, about as long as discal setae (Fig. 8G)	***Haplothripsalpester*** [part]
–	Anteromarginal setae long, at least twice as long as discal setae (cf. Fig. 7G)	***Haplothripstritici*** [part]
28 (8)	Fore femora with apical teeth (Fig. 9A); 3 sense cones on antennal segment III–IV (Fig. 9B)	***Acanthothripsnodicornis***
–	Fore femora without apical teeth; if teeth are present then antennal segment IV with 4 sense cones	**29**
29 (28)	Eyes ventrally elongated (Fig. 9C)	***Cephalothripsmonilicornis***
–	Eyes not ventrally elongated	**30**
30 (29)	Mouth cone long and pointed, extending beyond posterior margin of pronotum (Fig. 9D)	***Poecilothripsalbopictus***
–	Mouth cone shorter	**31**
31 (30)	Antennal segment III without sense cones (Fig. 9E)	***Lispothripscrassipes***
–	Antennal segment III with at least 1 sense cone (Fig. 9F)	**32**
32 (31)	Antennal segment III with 1 sense cone	**33 (*Liothrips*)**
–	Antennal segment III with 2 or 3 sense cones (Fig. 9F)	**34**
33 (32)	Setae S1 on abdominal tergite IX about as long as tube (Fig. 9G)	***Liothripsaustriacus***
–	Setae S1 on abdominal tergite IX about half as long as tube (Fig. 10A)	***Liothripssetinodis***
34 (32)	Abdomen clearly bicolored, with at least segment VIII–IX largely yellow (Fig. 10B); tube yellow but often with dark transverse terminal band or shading; micropterous forms usually with head and pronotum yellow	**35**
–	Abdomen uniformly brown or uniformly yellow, sometimes with pale or red markings	**36**
35 (34)	Abdominal segment VIII–X yellow (Fig. 10B)	***Hoplothripspedicularius***
–	Abdominal segment VI–X yellow	***Hoplothripscaespitis*** [part]
36 (34)	Pronotum with 5 pairs of well-developed setae, sometimes short but stout (Fig. 10C)	**37**
–	Pronotum with 4 pairs of well-developed setae, anteromarginals not distinctly stouter than discal setae	**43**
37 (36)	Postocular setae present but short, shorter or as long as width of eyes (Fig. 10D); setae S1 on abdominal tergite IX distinctly shorter than half of the length of tube (Fig. 10F)	**38 (*Phlaeothrips*)**
–	Postocular setae well developed and as long as or longer than the length of eyes (Fig. 10E); setae S1 on abdominal tergite IX at least half as long as tube (Fig. 10G)	**41**
38 (37)	Head with lateral tubercles (Fig. 10H)	**39**
–	Head without lateral tubercles (Fig. 10D)	**40**
39 (38)	Antennal segment III about 3 times as long as wide. Tibia I often completely yellow (Fig. 10I)	***Phlaeothripscoriaceus***
–	Antennal segment III less than 2.6 times as long as wide. Tibia I usually yellow apically (Fig. 10J)	***Phlaeothripsdenticauda***
40 (38)	Fore tibiae largely yellow, mid and hind tibiae distinctly bicolored with yellow apex and base (Fig. 10K)	***Phlaeothripsannulipes***
–	All tibiae brown	***Phlaeothripsbispinosus***
41 (37)	Postocular setae (Fig. 10E) and setae S1 on abdominal tergite IX expanded apically (Fig. 10G). Fore wings constricted medially	***Hoplandrothripsbidens***
–	Postocular setae and setae S1 on abdominal tergite IX acute. Fore wings parallel sided	**42**
42 (41)	Large pronotal setae expanded apically (Fig. 11A)	***Holothripsschaubergeri***
–	Large pronotal setae acute	***Hoplothripspolysticti*** [part]
43 (36)	Antennal segment IV with 2 sense cones (Fig. 9F)	**44**
–	Antennal segment IV with 3 or 4 sense cones	**48**
44 (43)	Maxillary stylets about 1/3 of head width apart (Fig. 11B)	**45**
–	Maxillary stylets close together, meeting or almost meeting medially (Fig. 4B)	**46**
45 (44)	Large pronotal setae expanded apically (cf. Fig. 11A). Antennal segments VII and VIII broadly attached	***Hoplothripslongisetis***
–	Pronotal setae acute	***Hoplothripscaespitis*** [part]
46 (44)	Setae S1 as long as or longer than tube (Fig. 11C)	***Hoplothripsunicolor*** [part]
–	Setae S1 shorter than tube	**47**
47 (46)	Antennal segment I slightly tapering apically, apical width less than 40 microns (Fig. 11D). Macropterous females with clusters of small sense cones on antennal segments IV–V (Fig. 11D)	***Hoplothripssemicaecus*** [part]
–	Antennal segment I more evenly tubular, apical width more than 40 microns	***Hoplothripscarpathicus***
48 (43)	Antennal segment IV with 3 sense cones	**49**
–	Antennal segment IV with 4 sense cones	**52**
49 (48)	Macropterous females with cluster of small sense cones on antennal segments IV–V (Fig. 11D). Males with small eyes, abdominal sternite VIII with irregularly and broadly shaped glandular pore area on sternite VIII	***Hoplothripssemicaecus*** [part]
–	Antennal segments different. Males without consciously small eyes, if small then with a defined circular glandular pore area on abdominal sternite VIII (Fig. 11E)	**50**
50 (49)	Setae S1 on abdominal tergite IX blunt apically (Fig. 11F)	***Thorybothripsunicolor***
–	Setae S1 on tergite abdominal IX acute (Fig. 11G)	**51**
51 (50)	Setae S1 on abdominal tergite IX as long as or longer than tube (Fig. 11C)	***Hoplothripsunicolor*** [part]
–	Setae S1 on abdominal tergite IX shorter than tube (Fig. 11G)	***Hoplothripspolysticti*** [part]
52 (48)	Major pronotal setae expanded (cf. Fig. 11A)	***Hoplandrothripswilliamsianus***
–	Major pronotal setae acute or blunt, not expanded	**53**
53 (52)	Antennal segment III asymmetric with long and strongly inwards curving sense cone at inner margin (Fig. 11H)	***Hoplothripsfungi***
–	Sense cones on segment III forwardly pointing and stout	**54**
54 (53)	Antennal segments IV–VI brown, IV at most slightly shaded (Fig. 11D)	**55**
–	Antennal segments IV–VI bicolored with basal half yellow (Fig. 11I)	**56**
55 (54)	Antennal segment VIII not distinctly constricted at base, VII and VIII confluent (Fig. 11D)	***Hoplothripssemicaecus*** [part]
–	Antennal segment VIII constricted at base, separating VII and VIII (Fig. 11J)	***Hoplothripspolysticti*** [part]
56 (54)	All tibiae completely yellow (Fig. 11K)	***Hoplothripscorticis***
–	Only fore tibia yellow, mid and hind tibiae at most yellow basally and apically (Fig. 11L)	***Hoplothripsulmi***

### ﻿Checklist of the Swedish Phlaeothripidae

**Idolothripinae Bagnall, 1908**

**Diagnosis.** The Idolothripinae are distinguished by the broad maxillary stylets. The maxillary stylets are at least 5 μm broad.

**Notes.** There are seven known species in Sweden in this subfamily. The broad maxillary stylets are hypothesized to be an adaptation to feeding on fungal spores (Mound and Palmer 1983).

#### *Bacillothrips* Buffa, 1908

***Bacillothripsnobilis* (Bagnall, 1909)**

Figs 4C, 5C, D

**Distribution.**Go.

**Remarks.** First record for Sweden. In Fennoscandia this species has previously been recorded from Denmark, Norway, and Finland (Kobro 2011; Gertsson 2015b). Feeding on fungal spores (Mound 1974), and found in dry grass, sedges, and on dead branches mainly from *Salix* L. (Mound et al. 1976; Schliephake and Klimt 1979).

**Material examined.** Sweden • 1♀; Gotland, Gotlands kommun, Vitärtskällan; 57.8512°N, 18.8123°E; 10 Jul. 2011; B. Eklund, leg.; Malaise trap; Loc. 029-06.

#### *Bolothrips* Priesner, 1926

***Bolothripsbicolor* Heeger, 1852**

**Distribution.**Up.

***Bolothripsdentipes* (Reuter, 1880)**

Fig. 5A

**Distribution.**Sk, Sm, Öl, Ög, Bo, Sö, Up, Lu.

***Bolothripsicarus* (Uzel, 1895)**

Fig. 4A

**Distribution.**Sk, Öl, Go, GS, Sö, Up.

**Remark.** First record for Sö.

**Material examined.** Sweden • 1♀; Södermanland, Nyköping kommun, Skeppsvik; dry meadow at roadside with *Crepis*, *Vicia*, and *Plantago*; 58.6399°N, 16.8225°E; 3 Jun. 2021; E. Wahlberg, leg.

#### *Cryptothrips* Uzel, 1895

***Cryptothripsnigripes* (Reuter, 1880)**

Fig. 4D

**Distribution.**Sk, Sm, Öl, Bo, Sö, Up, Vs, Vr, Dr, Lu.

#### *Megathrips* Targioni-Tozzetti, 1881

***Megathripslativentris* (Heeger, 1852)**

Fig. 5F

**Distribution.**Sk, Bl, Sm, Öl, Go, GS, Ög, Vg, Bo, Ds, Nä, Sö, Up, Vr, Dr, Gä, Hs, Me, Hr, Jä, Ån, Vb, Nb, Ly, Pi, Lu, To.

#### *Megalothrips* Uzel, 1895

***Megalothripsbonanni* Uzel, 1895**

Fig. 5E

**Distribution.**Sk.

**Phlaeothripinae Uzel, 1895**

**Diagnosis.** Differentiated from Idolothripinae by the slender maxillary stylets, at most 3 microns wide.

**Notes.** The majority of phlaeothripids belongs to this subfamily; from Sweden 42 species are known. The life histories are very varying, ranging from species feeding on fungal hyphae to predatory species (Mound and Tree 2020).

#### *Acanthothrips* Uzel, 1895

***Acanthothripsnodicornis* (Reuter, 1880)**

Fig. 9A, B

**Distribution.**Sm, Bo, Sö, Up, Vs, Vr, Dr.

#### *Cephalothrips* Uzel, 1895

***Cephalothripsmonilicornis* (Reuter, 1880)**

Fig. 9C

**Distribution.**Sk, Vg, Öl, Sm, Sö, Up, Vr.

**Remark.** First record for Vg.

**Material examined.** Sweden • 2♀♀; Västergötland, Laxå kommun, Finnerödja; sandy slope with *Carex* and *Calluna*; 58.9297°N, 14.3400°E; 5 Jun. 2021; E. Wahlberg, leg.

#### *Haplothrips* Amyot & Serville, 1843

***Haplothripsacanthoscelis* (Karny, 1910)**

Fig. 8C

**Distribution.**Sk, Öl.

***Haplothripsaculeatus* (Fabricius, 1803)**

Fig. 7B

**Distribution.**Sk, Bl, Ha, Sm, Öl, Go, Ög, Vg, Bo, Sö, Up.

**Remarks.** First record for Ha.

**Material examined.** Sweden • 1♀; Halland, Varberg kommun, Tvååker; meadow on old cultivated land with *Quercus*, *Fagus*, and *Fraxinus*; 57.0208°N, 12.4795°E; 19 May 2021; E. Wahlberg, leg.

***Haplothripsalpester* Priesner, 1914**

Figs 6E, 7A, 8G

**Figure 6.**
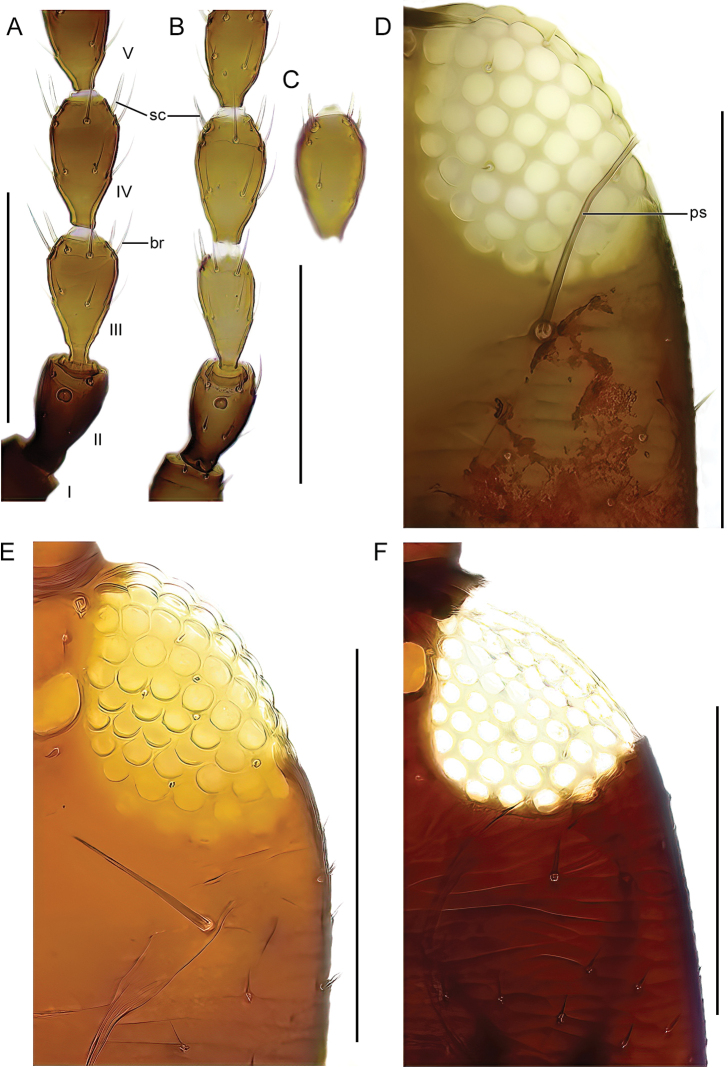
**A–C** part of antenna, dorsal view, I–VI: segment number **A***Xylaplothripsfuliginosus***B***Haplothripssubtilissimus***C** segment IV, ventral view, *H.subtilissimus***D–F** dorsal view of right side of head **D***H.subtilissimus***E***H.alpester***F***H.leucanthemi*. Abbreviations: sc: sense cones, br: bristle, ps: postocular setae. Scale bars: 100 μm.

**Distribution.**Sk, Öl, Vg, Sö, Vr, Ly.

**Remarks.** First record for Sö. This species is variable in the number of sense cones on segment III.

**Material examined.** Sweden • 1♂; Södermanland, Nyköping kommun, Skeppsvik; marsh with *Hierochloë*, *Juncus*, *Carex*, *Luzula* and *Schoenoplectus*; 58.6456°N, 16.8431°E; 3 Jun. 2021; E. Wahlberg, leg.

***Haplothripsalpicola* Priesner, 1950**

Fig. 7G

**Figure 7.**
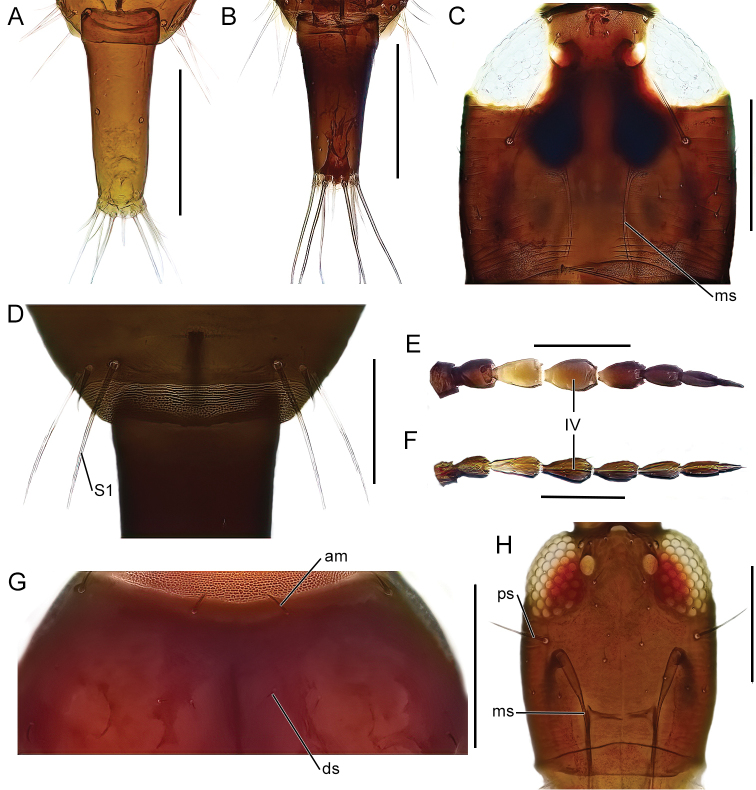
**A, B** dorsal view of tube **A***Haplothripsalpester***B***H.aculeatus***C** head, dorsal view, *H.statices***D** tergite IX, dorsal setae, *H.leucanthemi***E, F** antennae, dorsal view, antennal segment IV marked **E***H.leucanthemi***F***H.propinquus***G** part of pronotum, dorsal view, *H.alpicola***H** head dorsal view, *H.tritici*. Abbreviations: ms: maxillary stylets, S1: setae 1, am: anteromarginal setae, ds: discal setae, ps: postocular setae. Scale bars: 100 microns.

**Distribution.**Ly.

***Haplothripsangusticornis* Priesner, 1921**

**Distribution.**Sk, Ög, Up, Vr.

***Haplothripsdistinguendus* (Uzel, 1895)**

**Distribution.**Sk, Sm, Vg, Up.

***Haplothripshukkineni* Priesner, 1939**

Fig. 8E

**Figure 8.**
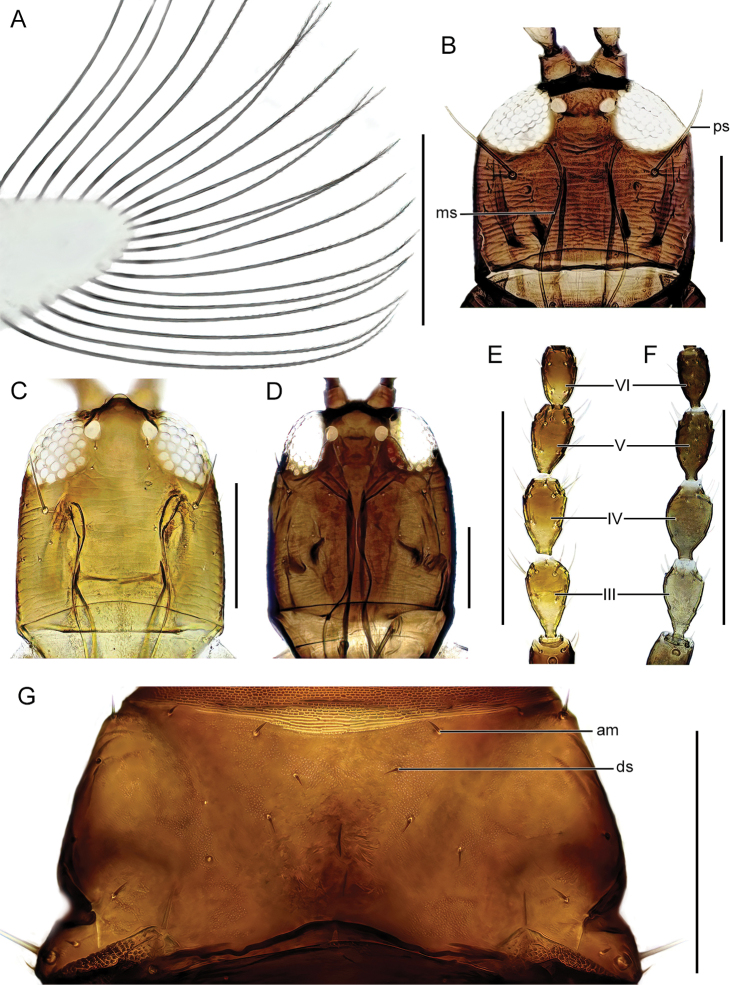
**A** distal portion of fore wing, *Haplothripssetiger***B–D** dorsal view of head **B***H.verbasci***C***H.acanthoscelis***D***H.utae***E, F** antennae, III–VI: segment number **E***H.hukkineni***F***H.tritici***G** pronotum, dorsal view, *H.alpester*. Abbreviations: ps: postocular setae, ms: maxillary stylets, am: anteromarginal setae, ds: discal setae. Scale bars: 100 μm.

**Distribution.**Sk, Sm, Öl, GoSö, Up, Vr.

***Haplothripsleucanthemi* (Schrank, 1781)**

Figs 1, 6F, 7D–E

**Distribution.**Sk, Ha, Sm, Öl, Bo, Ds, Nä, Ög, Sö, Up, Vr, Jä, Vb, Lu, To.

**Remark.** First record for Ha.

**Material examined.** Sweden • 1♂; Halland, Halmstad kommun, Särdal; on *Armeriamaritima*; 56.7367°N, 12.6472°E; 19 May 2021; E. Wahlberg, leg.

***Haplothripsminutus* (Uzel, 1895)**

**Distribution.**Sk, Sö.

***Haplothripspropinquus* Bagnall, 1933**

Fig. 7F

**Distribution.**Sk, Sö, Up, Vr, Gä, Hs, Jä, Lu.

***Haplothripssenecionis* Bagnall, 1932**

Fig. 5B

**Distribution.**Öl.

***Haplothripssetiger* Priesner, 1921**

Fig. 8A

**Distribution.**Sk.

***Haplothripsstatices* (Haliday, 1836)**

Fig. 7C

**Distribution.**Sk, Bl, Ha, Sm, Öl, Ög, Bo, Sö, Up, Hs, Hr, Jä, Ån, Nb, Lu.

***Haplothripssubtilissimus* (Haliday, 1852)**

Fig. 6B–D

**Distribution.**Sk, Sm, Sö, Up.

**Remark.** First record for Sm.

**Material examined.** Sweden • 1♀; Småland, Kalmar kommun, Bottorp; alley with *Quercus* and *Prunus*; 56.591923°N, 16.212710°E; 11 May 2021; E. Wahlberg, leg.

***Haplothripstritici* (Kurdjumov, 1912)**

Figs 7H, 8F

**Distribution.**Sö.

**Remark.** Setae variable in shape.

***Haplothripsutae* Klimt, 1970**

Figs 3, 8D

**Distribution.**Sk, Sm.

***Haplothripsverbasci* Osborn, 1896**

Fig. 8B

**Figure 9.**
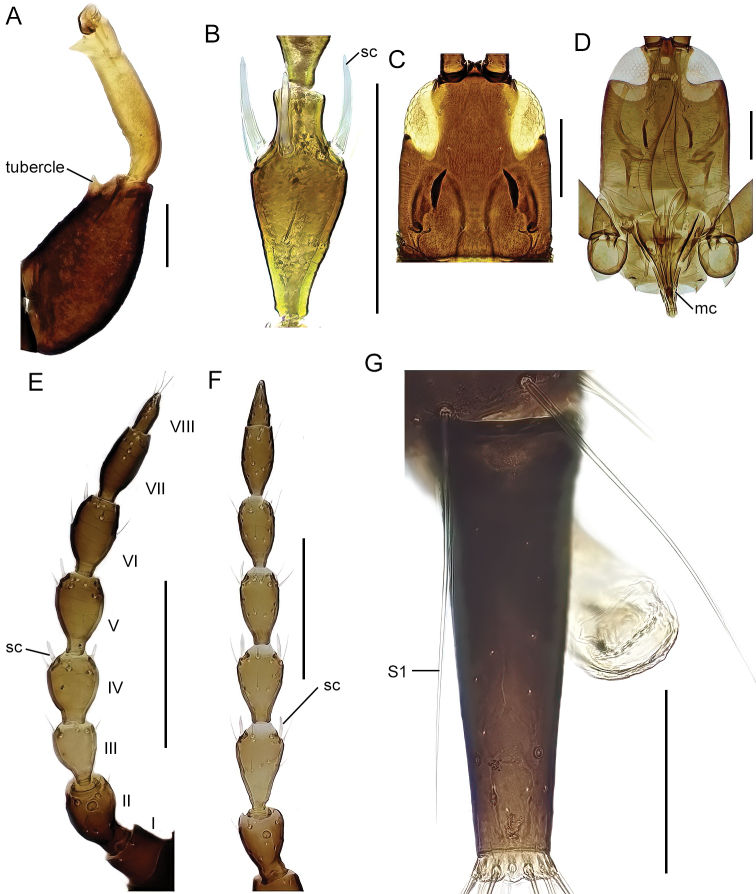
**A** fore leg, *Acanthothripsnodicornis***B** antennal segment IV, *A.nodicornis***C** head, ventral view, *Cephalothripsmonilicornis***D** head and pronotum with mouth cone (mesonotum detached), *Poecilothripsalbopictus***E, F** antennae, I–VIII: segment number **E***Lispothripscrassipes***F***Hoplothripslongisetis***G** abdominal segments IX–X, parts of the protruding phallus visible right side under the tube, *Liothripsaustriacus*. Abbreviations: sc: sense cones, mc: mouth cone, S1: setae 1. Scale bars: 100 μm.

**Distribution.**Sk.

#### *Holothrips* Karny, 1911

***Holothripsschaubergeri* (Priesner, 1920)**

Fig. 11A

**Distribution.**Sö.

#### *Hoplandrothrips* Hood, 1912

***Hoplandrothripsbidens* (Bagnall, 1910)**

Fig. 10E, G

**Figure 10.**
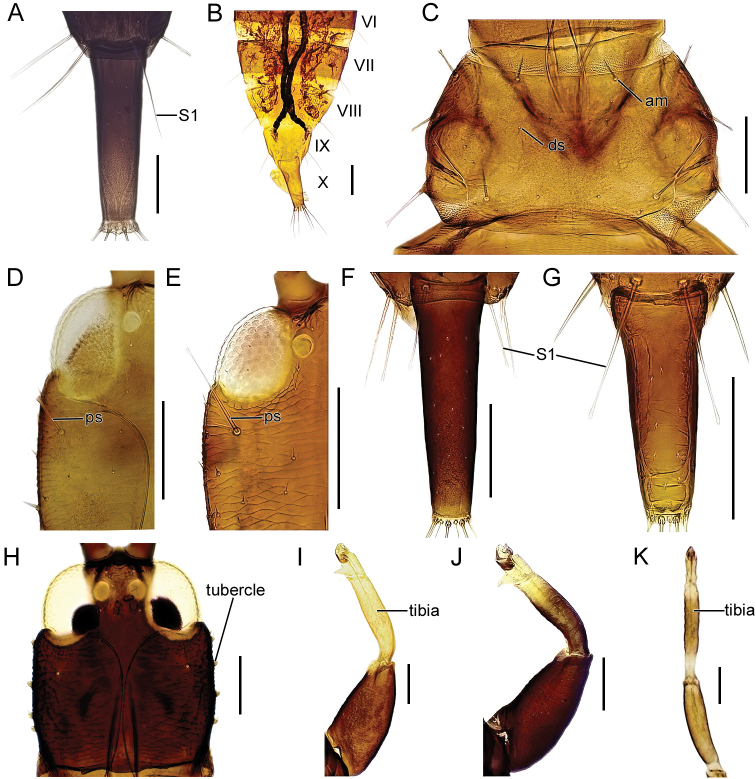
**A** abdominal segments IX–X, *Liothripssetinodis***B** abdomen, dorsal view, VI–X: segment number, *Hoplothripspedicularius***C** pronotom, dorsal view, *Phlaeothripsannulipes***D–E** left half of head, dorsal view **D***P.annulipes***E***Hoplandothripsbidens***F, G** abdominal segments IX (setae) and X **F***P.annulipes***G***H.bidens***H** head, dorsal view, *Phlaeothripscoriaceus***I–K** fore legs, dorsal view **I***P.coriaceus***J***P.denticauda***K** mid leg, *P.annulipes*. Abbreviations: S1: setae 1, am: anteromarginal setae, ds: discal setae, ps: postocular setae. Scale bars: 100 μm.

**Distribution.**Sk, Öl, Up.

***Hoplandrothripswilliamsianus* Priesner, 1923**

**Distribution.**Vr.

#### *Hoplothrips* Amyot & Serville, 1843

***Hoplothripscaespitis* (Uzel, 1895)**

**Distribution.**Sk.

**Remarks.** This species is variable in body colour, occurring in both bicolored and completely brown forms.

***Hoplothripscarpathicus* Pelikán, 1961**

Fig. 4B

**Distribution.**Sk, Ds, Sö, Up, Vr.

***Hoplothripscorticis* (de Geer, 1773)**

Fig. 11K

**Figure 11.**
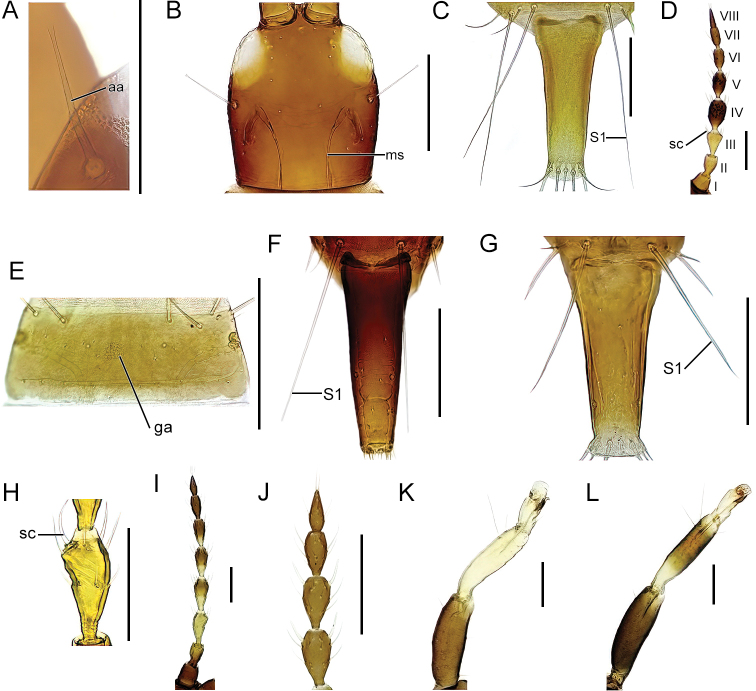
**A** anterolateral portion of pronotum, dorsal view, *Holothripsschaubergeri***B** head, dorsal view, *Hoplothripslongisetis***C** segments IX (setae) and X, *H.unicolor***D** antennae, I–VIII: segment number, *H.semicaecus***E** abdominal sternite VIII, *H.unicolor***F, G** abdominal segments IX–X **F***Thorybothripsunicolor***G***Hoplothripspolysticti***H** antennal segment III, *H.fungi***I** antenna, *H.ulmi***J** antennal segments V–VIII, *H.polysticti***K, L** mid leg **K***H.corticis***L***H.ulmi*. Abbreviations: aa: anteroangular setae, ms: maxillary stylets, sc: sense cones, ga: glandular pore area. Scale bars: 100 μm.

**Distribution.**Sk, Bl, Öl, Go, GS, Sm, Ög, Bo, Sö, Up, Vb, Nb.

**Remark.** First record for Nb.

**Material examined.** Sweden • 1♀; Norrbotten, Åsele, Björnlandet national park; 63.9702°N, 18.0533°E; 12–26 Jul. 2011; K. Norberg, B.O. Johansson, leg.; Malaise trap; Loc. 034-04.

***Hoplothripsfungi* (Zetterstedt, 1828)**

Fig. 11H

**Distribution.**Bl, Öl, Go, Up, Hs.

***Hoplothripslongisetis* (Bagnall, 1910)**

Figs 9F, 11B

**Distribution.**Sk, Ds, Vr.

***Hoplothripspedicularius* (Haliday, 1836)**

Fig. 10B

**Distribution.**Sk, Sm, Sö, Up, Vr, Dr, Hs.

***Hoplothripspolysticti* (Morison, 1949)**

Fig. 11G, J

**Distribution.**Sk, Ög, Vr, Dr, Vb.

**Remarks.** This species is variable in the number of sense cones on both antennal segments III and IV, as well as in number of large pronotal seatae.

***Hoplothripssemicaecus* (Uzel, 1895)**

Fig. 11D

**Distribution.**Sk, Ha, Up.

**Remarks.** First record for Ha. Very variable in the number of sense cones on both antennal segments III and IV, as well as many structural differences in macropterous and apterous males and females.

**Material examined.** Sweden • 3♂♂; Halland, Falkenberg kommun, Vessigebro; deciduous forest (*Fagus*), in *Fomesfomentarius*; 57.0575°N, 12.7888°E; 18 May 2021; E. Wahlberg, leg.

***Hoplothripsulmi* (Fabricius, 1781)**

Fig. 11I, L

**Distribution.**Sk, Bl, Ha, Sm, Öl, Go, GS, Ög, Bo, Ds, Sö, Up, Vs, Vr, Dr, Ån, Vb, Lu.

***Hoplothripsunicolor* (Vuillet, 1914)**

Fig. 11C, E

**Distribution.**Sö.

**Remark.** This species is variable in the number of sense cones on antennal segment IV.

#### *Liothrips* Uzel, 1895

***Liothripsaustriacus* (Karny, 1910)**

Fig. 9G

**Distribution.**Vr.

***Liothripssetinodis* (Reuter, 1880)**

Fig. 10A

**Distribution.**Ha, Up.

#### *Lispothrips* Reuter, 1899

***Lispothripscrassipes* (Jablonowski, 1894)**

Fig. 9E

**Distribution.**Sm.

***Phlaeothripsannulipes* Reuter, 1880**

Fig. 10C, D, F, K

**Distribution.**Sk, Sm, Ög, Bo, Sö, Up, Vs, Vr, Dr, Vb.

***Phlaeothripsbispinosus* Priesner, 1919**

**Distribution.**Vr.

***Phlaeothripscoriaceus* Haliday, 1836**

Fig. 10H, I

**Distribution.**Sk, Bl, Ha, Sm, Öl, Go, Bo, Sö, Up, Vs, Hs, Vb.

***Phlaeothripsdenticauda* Priesner, 1914**

Fig. 10J

**Distribution.**Sk, Vr.

#### *Poecilothrips* Uzel, 1895

***Poecilothripsalbopictus* Uzel, 1895**

Fig. 9D

**Distribution.**Sk, Vr.

#### *Thorybothrips* Priesner, 1924

***Thorybothripsunicolor* (Schille, 1911)**

Fig. 11F

**Distribution.**Öl, Go.

#### *Xylaplothrips* Priesner, 1928

***Xylaplothripsfuliginosus* (Schille, 1911)**

Fig. 6A

**Distribution.**Sm, Ha, Sö, Ds, Vr, Dr, Lu, To.

**Remark.** First record for Ha and Sö.

**Material examined.** Sweden • 1♂; Halland, Falkenberg kommun, Vessigebro; on *Larixdecidua*, 56.9748°N, 12.7288°E, 19 May 2021; E. Wahlberg, leg. • 2♀♀; Södermanland, Nyköping kommun, Skeppsvik; mixed forest; 58.6458°N, 16.8431°E; 03 Jun. 2021; E. Wahlberg, leg. • 1♀; Södermanland, Gnesta kommun, Fridsta; private garden with mixed vegetation; 59.0673°N, 17.1550°E; 14–21 Jun. 2021; E. Wahlberg, leg., window trap. • 1♂ Södermanland, Gnesta kommun, Önnersta; on dead *Betula*; 59.0470°N, 17.1460°E; 16 Jul. 2021; E. Wahlberg, leg.

## Supplementary Material

XML Treatment for Phlaeothripidae
